# Working Memory Depletion Affects Intertemporal Choice Among Internet Addicts and Healthy Controls

**DOI:** 10.3389/fpsyg.2021.675059

**Published:** 2021-12-17

**Authors:** Hongxia Li

**Affiliations:** School of Labor Economics, Capital University of Economics and Business, Beijing, China

**Keywords:** addictive behavior, intertemporal decision making, delay discounting rate, subjective value, internet addicts

## Abstract

Addiction to the Internet has emerged as a new kind of addictive behavior. Although previous studies have revealed that impairments in working memory led to suboptimal decision making (e.g., a greater willingness to choose smaller, more immediate rewards), little is known about how working memory affects intertemporal choice in Internet addicts and normal users. Thus, this study’s aim was to investigate the effect of working memory task on intertemporal choice in 33 participants addicted to internet and 25 healthy controls. Participants were administered (a) a test for Internet Addiction, (b) a single delay discounting self-report questionnaire (c) a working memory task. Differences between the Internet addicts and the control group were observed in terms of delay discounting rates, reaction times, and in memory accuracy rates. We observed significantly higher delay discounting rates among individuals addicted to the Internet. Moreover, it was documented that reaction times follow the 4-level working memory condition were significantly longer than follow the 2-level condition, in both the Internet addicts and the control group. The current findings suggest that Internet addicts are more likely to make short-sighted decisions than normal Internet users. The higher the level of working memory, the more likely an individual is to choose the present smaller reward, thus making short-sighted decisions, and have longer response times.

## Introduction

Internet addiction has been known to be linked to a host of negative outcomes for those who are addicted to internet (Koronczai et al., 2011), such as reduced work efficiency (Anandarajan et al., 2000), lowered academic achievement and social relationship (Young, 1996; Young et al., 1999; Cash et al., 2012), increased abnormal behaviors and behavioral disorders (Lam et al., 2009; Lin et al., 2011; Li et al., 2016, 2019), and neurological dysfunction (Dalbudak et al., 2014; Petry et al., 2014, 2018). Studies have indicated that short-sighted decision making is related to cognitive function impairment among individuals with addictive disorders (Hare et al., 2009; Levitt et al., 2010; Cruz Rambaud et al., 2017; Calluso et al., 2020). Moreover, previous studies found that there is a significantly negative correlation between working memory and delay discounting rates (Hinson et al., 2003; Shamosh et al., 2008). Delay discounting rate is an indicator of intertemporal choice (Frederick et al., 2002). We think that the working memory plays an important role in intertemporal decision making.

According to hot-cold system model (Loewenstein, 1996) on intertemporal decision, the reason why individuals can make long-term decisions instead of indulging in current temptation is that they are guided by rationality for goal-oriented behaviors and effective willpower is additionally needed for behavior control. The result of intertemporal choice depends on whether the cold or the hot system prevails. The cold system achieves long-term self-monitoring through different executive functions, such as reasoning and judging work memory, so that individuals can resist behavior habits. The cold system is goal-oriented and related to the high self-control of individuals, reasonable self-interest and rational decision-making behaviors. On the contrary, the hot system make decisions based on intuitions without rational reasoning and deliberate thinking. The individuals follow the principle of perception, namely they will perform it as long as they feel good, and are often affected by emotion and intuition (Loewenstein, 1996; Metcalfe and Mischel, 1999). According to the distinction between hot and cold systems, working memory, being a cold process, can directly affect individual’s intertemporal decision making performance. What different levels of difficulty in working memory task affect intertemporal decision making, however, remains unknown.

Working memory has been identified as an important component of the central executive, whose principal function is the supervision of information entered, where older information that is less relevant to the task at hand is replaced by larger, newer, more relevant information; thus, the contents of memory are constantly being amended (Baddeley and Hitch, 1974; Morris and Jones, 1990; Collette and Van der Linden, 2002; Bledowski et al., 2006, 2009; Baddeley, 2007, 2012; Palladino and Artuso, 2018). We argue that working memory as an integral aspect of executive functioning can explain short-sighted decision-making behaviors in intertemporal choice. Working memory is often used to store limited information that is presently the focus of attention. Because individuals need to make comparisons between both options or outcomes and integrate relevant information when making intertemporal decisions, such evaluations based on working memory and exhaust more cognitive resources. The existence of a high correlation between damage to the executive function of working memory in individuals has previously been reported (Whitney et al., 2004). Research conducted on working memory training provides further support for the argument that working memory is a critical factor in intertemporal decision making. Bickel et al. (2011b) conducted an experiment involving stimulant addicts, who were each assigned to either the training group or control group. Significant decreases were observed in the delay discounting rates of the participants who received working memory training (i.e., the training group) as compared to those who did not (i.e., the control group). Furthermore, there was a significant positive correlation between working memory and delay discounting rates.

In the present study, we aimed to investigate how different levels of difficulty in working memory affect intertemporal decision making among Internet addicts and normal users. The delay discount rate represents individual’s impulsiveness in the intertemporal choice, and the higher the delay discount rate is, the more impulsiveness in the intertemporal choice (Cruz Rambaud and Muñoz Torrecillas, 2016; Thaler, 2016). We hypothesized that the delay discounting rates of Internet addicts were significantly higher than healthy controls. Furthermore, we hypothesized the delay discounting rates of both Internet addicts and healthy controls in the high-difficulty level working memory condition to be significantly higher than those of participants in the low-difficulty level working memory condition. Additionally, we hypothesized that working memory accuracy rates would be significantly lower among Internet addicts than in healthy controls, and that the reaction times of Internet addicts would be significantly longer than those of healthy controls.

## Materials and Methods

### Experimental Design

This study employed a 2 (participant type: Internet addicts vs. healthy controls) × 2 (working memory level of difficulty: 2-level vs. 4-level) completely randomized experimental design. The dependent variables examined in this study were the delay discounting rate in intertemporal decision making, working memory accuracy rates and reaction times. SPSS 20.0 software was used for data entry and variance analysis. The operational definitions for the dependent variables as follows:

The delay discounting rate. The delay discount rate represents the degree of short-sightedness in the intertemporal decision task, and the higher the delay discount rate is, the more short-sighted decision.

Working memory accuracy rates. Working memory accuracy refers to the correct number of working memory tasks divided by the total number of experiments.

Working memory reaction times. Working memory response time refers to the time from the presence of stimulus to the response of the subject in the process of working memory task.

### Participants

Thirty-three participants (15 female, 18 males; mean age = 17 years) being treated for Internet addiction at an Internet addiction withdrawal school in Beijing and twenty-five healthy controls (18 female, 7 males; mean age = 18 years) from Beijing Union University were recruited to participate in this study. We used Young’s (1999) assessment scale to ensure that all selected Internet addicts met the diagnostic criteria for Internet addiction. Internet addicts and normal Internet users were well matched in terms of age. All participants had never participated in an experiment similar to the present one. We excluded subjects who had keep picking A, or B 100% of trials in the intertemporal choice tasks. We eliminated 2 Internet addicts and 3 healthy controls. So, the participants and data analysis results reported are based on this criterion. Additional participant characteristics are presented in Table 1.

**TABLE 1:** Characteristics of participants (*M* ± *SD*).

Participant group	Working memory	*n*	Age	Education years	Addiction scores
Normal users	2-level	13	18.10 ± 1.79	11.35 ± 0.81	24.90 ± 2.79
	4-level	12	18.86 ± 1.04	11.08 ± 0.65	24.84 ± 2.87
Internet addicts	2-level	18	18.20 ± 2.32	10.26 ± 1.15	48.00 ± 8.69
	4-level	15	17.62 ± 1.68	11.00 ± 1.48	47.80 ± 8.44

This study was reviewed and approved by the Committee of Protection of Subjects at Beijing Normal University. All participants provided written informed consents before the study, and were fully debriefed at the end of the research, according to guidelines established by the committee. All participants received a small gift at the conclusion of the experiment.

### Materials

#### The Internet Addiction Test

The Internet Addiction Scale, formulated by Young (1999), has been used to reliably and validly assess addictive use of the Internet worldwide. This instrument consists of 20 items rated on a 5-point Likert-type scale, with 1 = *rarely* and 5 = *always*. Respondents’ total scores on the scale are calculated to diagnose their degree of addiction to the Internet, with higher scores signifying a greater degree of addiction. Scores less than 49 signify average Internet use: although these individuals might surf the Web a bit too long at times, they still have control over their usage. Scores exceeding 49, however, indicate Internet addiction. A Cronbach’s alpha coefficient of 0.9085 and split-half reliability coefficient of 0.8258 indicate that the scale’s Chinese version has excellent reliability and validity (Cao et al., 2011).

#### Delay Discounting Tasks

We utilized the delay discounting task comprising 19 binary-choice items (with A representing current options and B representing “6 months after” options) developed by Chen and He (2011). The total monetary reward for future options (Choice B) was fixed at 1,000 RMB, while that for current options ranged from a possible 50–950 RMB, with respondents standing to receive 50 RMB for every time they chose A, as outlined below:

“Imagine a situation in which you have a choice for either receiving less money right now, or a larger amount 6 months later. Which would you choose?

(1) A: Get 50 RMB (US$7.90) now; B: Wait for 6 months to get 1,000 RMB (US$158).
(2) A: Get 100 RMB (US$15.80) now; B: Wait for 6 months to get 1,000 RMB (US$158).
(3) A: Get 150 RMB (US$23.70) now; B: Wait for 6 months to get 1,000 RMB (US$158).
(4) A: Get 200 RMB (US$31.60) now; B: Wait for 6 months to get 1,000 RMB (US$158).
(5) A: Get 250 RMB (US$39.50) now; B: Wait for 6 months to get 1,000 RMB (US$158).
(6) A: Get 300 RMB (US47.40) now; B: Wait for 6 months to get 1,000 RMB (US$158).
(7) A: Get 350 RMB (US$55.30) now; B: Wait for 6 months to get 1,000 RMB (US$158).
(8) A: Get 400 RMB (US$63.20) now; B: Wait for 6 months to get 1,000 RMB (US$158).
(9) A: Get 450 RMB (US$71.10) now; B: Wait for 6 months to get 1,000 RMB (US$158).
(10) A: Get 500 RMB (US$79) now; B: Wait for 6 months to get 1,000 RMB (US$158).
(11) A: Get 550 RMB (US$86.90) now; B: Wait for 6 months to get 1,000 RMB (US$158).
(12) A: Get 600 RMB (US$94.80) now; B: Wait for 6 months to get 1,000 RMB (US$158).
(13) A: Get 650 RMB (US$102.71) now; B: Wait for 6 months to get 1,000 RMB (US$158).
(14) A: Get 700 RMB (US$110.61) now; B: Wait for 6 months to get 1,000 RMB (US$158).
(15) A: Get 750 RMB (US$118.71) now; B: Wait for 6 months to get 1,000 RMB (US$158).
(16) A: Get 800 RMB (US$126.41) now; B: Wait for 6 months to get 1,000 RMB (US$158).
(17) A: Get 850 RMB (US$134.51) now; B: Wait for 6 months to get 1,000 RMB (US$158).
1(8) A: Get 900 RMB (US$142.21) now; B: Wait for 6 months to get 1,000 RMB (US$158).
(19) A: Get 950 RMB (US$150.11) now; B: Wait for 6 months to get 1,000 RMB (US$158).”

The researchers found that the current subjective value (V) of the subjects to the delay reinforcer (A) at A certain delay time point (D) could be obtained after A certain number of choices of reinforcers of different magnitudes at different delay time points. The following formula (1) can be used to calculate the delay discounting rates (K). The higher the discounting rate is, the stronger the subject’s impulsivity is during decision making (Kirby et al., 1999).


(1) $$V=\frac{A}{1+k\,D}$$

This formula indicates that the current subjective value (V) and the subject’s discount rate (K) for the delay reinforcer are interchangeable. If the current subjective value is high, the subject’s decision impulsivity is low and the delay discount rate is low. On the contrary, if the current subjective value is low, the subject’s decision impulsivity is high, and the delay discount rate is high. In this study, we used the delay discount rate as the dependent variable.

#### Working Memory Tasks

Working memory tasks developed by Sternberg (1966) were presented to participants on a computer screen, using E-Prime software, according to the requirements of the current experiment. Tasks of two levels of difficulty were administered: 2-level and 4-level working memory tasks. A total of 30 experimental trials were conducted for each task level, with 20 trials seeking correct responses from participants and 10 seeking incorrect responses. Random digital numbers ranging from 0 to 9 appeared on screen in each trial—a different one each time. Two digital numbers were presented in sequence for each 2-level task trial and 4-level task trial.

### Procedure

All the subjects were recruited separately to the university laboratory for the experiment. It takes about 30 min for each subject to complete this study. Before the experiment began, both the Internet addicts and healthy controls were independently assigned to two groups in which they would complete 2-level or 4-level working memory tasks. The research was composed of three main phases, the first of which entailed working memory task exercises; the second, working memory tasks; and the third, intertemporal choice tasks. Internet addicts and healthy controls were independently assigned to either the group completing 2-level or 4-level working memory tasks based on their gender and Internet addiction scores.

In the working memory task exercise session, participants were told the requirements of the experiment and how they could respond to stimuli presented in six additional experimental trials. We carefully explained the procedures of the experiment to participants, using a 2-level working memory task as an example, whereby the following instructions guiding participants were presented on the computer screen:

*You may see 2 digital numbers sequentially on the screen, followed by a target digital number after a beeping sound. You are asked to judge whether the target digital number appeared in the former 2 digital numbers. If you judge that the target digital number appeared in the former 2 numbers, you can press the “F” key; if you judge that the target digital number did not appear in the former 2 numbers, you can press the “J” key. If you understand the requirements of this experiment, you can press the spacebar to start this experiment.*

A blank screen appeared for 500 ms after the instructions disappeared. This was followed by a black fixation point on the center of the screen for 500 ms, and then another blank screen for 500 ms. The first digital number appeared on the center of the screen for a duration of 500 ms after the fixation point disappeared, followed by a blank screen for 500 ms. The next digital number then appeared, followed by a blank screen for 500 ms and a beeping sound. Then the red target digital number emerged for a period of 12,000 ms, during which participants judged whether it appeared in the former 2 digital numbers. If the participant responded, he or she automatically proceeded on to the next experimental trial. If the participant did not respond, feedback (i.e., “no response”) in red font appeared on the upper left corner of the screen for as long as 500 ms. There were two possible types of feedback that participants could receive upon making a judgment. If they responded correctly, the feedback “*correct answer*” appeared in red on the upper left corner of the screen for 2,000 ms. If they responded incorrectly, the feedback “*wrong answer*” emerged in red in the upper left corner of the screen, also for a duration of 2,000 ms. After the working memory task session, all participants completed the delay discounting task and items designed to collect information on demographic variables.

## Results

### Delay Discounting Rate as the Dependent Variable

The statistical analysis results are as follows (Note: The figures with individual-level data are not included because individual-level data were no longer available). A significant main effect of participant type was observed, *F*(1, 54) = 8.700, *p* = 0.005, η^2^ = 0.139, reflecting significantly higher delay discounting rates among Internet addicts (*M* = 3.944, *SD* = 0.590) than among healthy controls (*M* = 1.298, *SD* = 0.676). In addition, there was a significant main effect of working memory level of difficulty was detected, *F*(1, 54) = 4.137, *p* = 0.047, η^2^ = 0.097. The delay discounting rate in the 2-level condition (*M* = 1.572, *SD* = 0.861) was significantly lower than that in the 4-level condition (*M* = 3.256, *SD* = 0.802). However, there was no significant interaction was found between participant type and level of difficulty in working memory, *F*(1, 51) = 1.200, *p* = 0.279, η^2^ = 0.023. The descriptive statistics of these variables are shown in Table 2.

**TABLE 2:** Descriptive statistics for delay discounting rate according to participant type and level of difficulty in working memory task (*M* ± *SD*).

Type of participants	Level of working memory	*n*	Delay discounting rate
Healthy controls	2-level	13	1.18 ± 0.94
	4-level	12	1.53 ± 0.96
Internet addicts	2-level	18	2.44 ± 0.80
	4-level	15	5.44 ± 0.87

### Working Memory Accuracy Rate as the Dependent Variable

With the rate of correct responses on working memory tasks as the dependent variable, the statistical analysis results are as follows. The descriptive statistics for these variables are shown in Table 3. A significant main effect of participant type was detected, *F*(1, 54) = 4.797, *p* = 0.033, η^2^ = 0.082, indicating that the working memory accuracy rates of Internet addicts (*M* = 0.900, *SD* = 0.017) were significantly lower than were those of healthy controls (*M* = 0.957, *SD* = 0.020). There was no significant main effect of working memory level of difficulty, and the interaction between participant type and level of difficulty in working memory was non-significant, *F*s < 1.

**TABLE 3:** Descriptive statistics for accuracy rates and reaction times according to participant type and level of difficulty in working memory (*M* ± *SD*).

Type of participants	Level of working memory	*n*	Accuracy rate (%)	Reaction time (ms)
Healthy controls	2-level	13	0.95 ± 0.04	725.75 ± 151.09
	4-level	12	0.96 ± 0.41	770.71 ± 111.31
Internet addicts	2-level	18	0.93 ± 0.06	1001.52 ± 182.87
	4-level	15	0.86 ± 0.17	1150.75 ± 224.30

### Working Memory Reaction Time as the Dependent Variable

With the reaction time as the dependent variable, the statistical analysis results are as follows. A significant main effect of participant type was detected, *F*(1, 54) = 48.849, *p* = 0.000, η^2^ = 0.475, implying that the reaction times of Internet addicts (*M* = 1076.132, *SD* = 30.860) were significantly longer than those of healthy controls (*M* = 748.230, *SD* = 35.337). There was a significant main effect of working memory level of difficulty as well, *F*(1, 54) = 4.283, *p* = 0.043, η^2^ = 0.073, suggesting that reaction times in the 4-level working memory condition (*M* = 960.727, *SD* = 34.188) were significantly longer than were those in the 2-level working memory condition (*M* = 863.634, *SD* = 32.129). No significant interactions were found. The descriptive statistics for these variables are shown in Table 3.

## Discussion

These results demonstrated that working memory load affects intertemporal decision making in both Internet addicts and healthy individuals. Furthermore, the delay discounting rates of Internet addicts were significantly higher than those of healthy controls, consistent with previous findings (Bickel et al., 2008; Businelle et al., 2010; Bickel et al., 2011a; Saville et al., 2017; Cheng et al., 2021). Thus, tasks involving a greater degree of difficulty in working memory elicited higher delay discounting rates in intertemporal decision making among Internet addicts as well as normal users. These results concurred with those of other investigations (Kirby et al., 1999; Kane et al., 2001; Hinson et al., 2003; Shamosh et al., 2008).

The current findings indicate that impulse control disorders associated with intertemporal decision making are caused by deficits in working memory ability. The significantly lower accuracy rates observed among Internet addicts in this study, and their significantly longer reaction times, in comparison to those recorded for healthy controls, provide strong support for this.

Thus, the present study appears to provide evidence for the association between working memory load, Internet addictive behaviors, and intertemporal decision making. Our findings resonated with those of former studies reporting that shortsighted decision making tends to result from impaired cognitive functioning in addictive disorders (Hare et al., 2009; Levitt et al., 2010). Furthermore, obstacles in working memory contributed to shortsighted decision making in this study, as individuals ignored larger long-term rewards for more immediate, smaller ones. This was also noted in prior investigations (Hinson et al., 2003; Engle and Kane, 2004). Additional prevention research into working memory training could provide further support for our findings from other perspectives or points of view (Bickel et al., 2011b).

In this study, a close link between cognitive load in working memory and intertemporal choice was noted. This link suggests that working memory ability may serve as a new indicator for assessing impulsive disorders. Executive functioning can be improved through working memory training (Lavie et al., 2004). Overall, these results imply that working memory task training could be an effective intervention for decreasing impulsivity in a variety of addictive behaviors.

We did not find a significant interaction between working memory load and participant type in the present study, however, which could be easily explained by the statistical analyses we performed in this study. That is, regardless of whether participants were addicted to the Internet, the delay discounting rates follow the 4-level (working memory task) condition were significantly lower than follow the 2-level condition. Along with previous findings, the present results offer powerful evidence for significantly higher levels of impulsivity in individuals struggling with addiction (Petry, 2001; Alessi and Petry, 2003; Bickel et al., 2008; de Wit, 2009; Passetti et al., 2011). The present study on Internet addiction further enriches our understanding of the link between addictive behaviors and intertemporal decision making.

The results of the current study can be supported by some physiological basis research that working memory is responsible for myopic decision-making (Hinson et al., 2003; Fellows and Farah, 2004). A neuroimaging study noted that the cortices associated with working memory were activated during the process of intertemporal decision making (McClure et al., 2004). Some researchers have suggested that the parahippocampal gyrus plays a highly significant role in the preservation and retrieval of information in working memory (Luck et al., 2010). Lower fractional anisotropy values for white matter abnormalities have been noted in the right side of the hippocampus for Internet addicts. According to Engle and Kane (2004), the prefrontal cortex is likely to be the structural basis for working memory dysfunction for Internet addicts. Structural abnormalities in the internal capsule hinder and damage executive and memory functions (Levitt et al., 2010), and fractional anisotropy abnormalities in the left posterior limb affect the processing and conversion of visual information, resulting in damage to cognitive control functions (Levitt et al., 2010).

The results of this study also suggest several critical directions for future research. First, significant decreases in the delay discounting rates of stimulant addicts have been demonstrated in previous research with the introduction of working memory training (Bickel et al., 2011b). Hence, we propose that we can decrease impulsivity in Internet addicts through such training in working memory task. Second, the previous literature shows that risk preferences may vary accordingly to contextual factors and depend on the type of tasks used to measure them (Addessi et al., 2013). Previous study has documented those hypothetical responses with questionnaire may underestimate the propensity to respond impulsively compared to tasks that measure real responses. Using only one measure to assess intertemporal decision making should be at least acknowledged as a limitation of the study. So, more than one single task should be used to assess risk preferences in the future study. Third, the number of subjects selected in the current study is relatively small. The future study will increase the number of subjects and investigate the influence of working memory refresh on other types of decision task, such as the Game of Dice Task (Yao et al., 2014), be conducted.

In sum, working memory load can affect individuals’ intertemporal decision making. The greater the working memory load, the shorter sighted the decisions or choices exhibited by individuals. This is especially evident in Internet addicts. Decision-making disorders likely stem from impairments in working memory as it constitutes an integral part of the psychological system involved in decision making. Additionally, preventions leveraging the strengths of working memory task training may be employed to effectively decrease impulsivity associated with addiction.

## Data Availability Statement

The original contributions presented in the study are included in the article/supplementary material, further inquiries can be directed to the corresponding author/s.

## Ethics Statement

The studies involving human participants were reviewed and approved by the Ethics Committee of Beijing Normal University. Written informed consent to participate in this study was provided by the participants’ legal guardian/next of kin.

## Author Contributions

HL finished the experiment and wrote the manuscript.

## Conflict of Interest

The author declares that the research was conducted in the absence of any commercial or financial relationships that could be construed as a potential conflict of interest.

## Publisher’s Note

All claims expressed in this article are solely those of the authors and do not necessarily represent those of their affiliated organizations, or those of the publisher, the editors and the reviewers. Any product that may be evaluated in this article, or claim that may be made by its manufacturer, is not guaranteed or endorsed by the publisher.
